# Supplementary material for: Massively parallel reporter assay reveals promoter-, position-, and strand-specific effects in transcription start sites
Source: bioRxiv. 2025 Oct 14:2025.10.13.659964. Preprint. [Version 1] doi: 10.1101/2025.10.13.659964 (PMC12632999; doi:10.1101/2025.10.13.659964)
Supplement: Supplement 1 [file media-1.pdf]

Supplementary Data 1: Review of 146 published MPRA studies, annotated by the number of different promoter elements and plasmid configurations each fragment was tested with. List of studies was generated using the Pubmed search (("massively parallel reporter assay") NOT (Review[Publication Type]) NOT (Meta-Analysis[Publication Type])) on Aug. 29, 2025. Databases, protocols, meta-analyses, and analyses that do not create new data were removed.

PMID	DOI	Number of promoters tested alongside fragment	Number of positions (plasmid configurations) fragment was tested in
35298243	10.1126/science.abj5117	1	1
37413987	10.1016/j.cell.2023.06.007	1	1
34534445	10.1016/j.cell.2021.08.025	1	1
30033119	10.1016/j.stem.2018.06.014	3	1
37071996	10.1016/j.devcel.2023.03.020	1	1
27259153	10.1016/j.cell.2016.04.027	1	1
36705030	10.1161/CIRCULATIONAHA.122.061955	1	1
34650237	10.1038/s41588-021-00947-3	1	1
37387536	10.1111/tpj.16373	1	1
37953348	10.1038/s42003-023-05483-w	1	1
36156153	10.1093/nar/gkac806	1	1
31530582	10.1101/gr.247312.118	1	2
28204611	10.1093/nar/gkw942	1	1
37996647	10.1038/s41556-023-01296-5	1	1
30158147	10.1101/gr.231886.117	2	1
36646877	10.1038/s41593-022-01243-x	1	1
38407202	10.7554/eLife.89371	1	1
34849835	10.1093/g3journal/jkab404	1	1
33885362	10.7554/eLife.63713	1	1
37658059	10.1038/s41467-023-41081-4	1	1
29225039	10.1016/j.molcel.2017.11.014	1	1
38389303	10.1016/j.xhgg.2024.100279	1	1
31152051	10.1101/gr.242552.118	1	1
30451991	10.1038/nbt.4285	2	1
29410437	10.1038/s41467-018-02980-z	1	2
36777181	10.1016/j.xgen.2022.100234	1	2
37087538	10.1038/s41467-023-37960-5	1	1
29889606	10.1080/21541264.2018.1486150	1	1
36107770	10.1093/nar/gkac763	1	1
35534523	10.1038/s41598-022-11589-8	1	1
33357440	10.1016/j.celrep.2020.108531	1	1
38183988	10.1016/j.ajhg.2023.12.008	1	1
36763080	10.7554/eLife.71235	2	1
38365907	10.1038/s41598-024-54302-7	1	1
36192170	10.1101/gr.276863.122	1	1
28525990	10.1186/s12864-017-3785-4	1	1

34978147	10.1002/alz.12534	1	1
36555130	10.3390/ijms232415485	1	1
37868037	10.1016/j.xgen.2023.100404	1	1
33712590	10.1038/s41467-021-21854-5	1	1
33970899	10.1371/journal.pcbi.1008982	1	1
26713262	10.7717/peerj.1527	1	1
37492106	10.1016/j.xgen.2023.100330	1	1
39818206	10.1016/j.devcel.2024.12.038	1	1
35866592	10.1093/gbe/evac108	1	1
32103011	10.1038/s41467-020-14853-5	1	1
22371084	10.1038/nbt.2137	1	1
31631012	10.1016/j.stem.2019.09.010	1	1
38647082	10.1093/nar/gkae285	1	1
22371081	10.1038/nbt.2136	1	1
33849996	10.2337/db20-1087	1	1
32239644	10.15252/emmm.202012112	1	1
34489471	10.1038/s41467-021-25614-3	1	1
32426415	10.1016/j.omtm.2020.04.006	1	1
34662402	10.1093/molbev/msab304	1	1
33626337	10.1016/j.ajhg.2021.02.006	1	1
23512712	10.1101/gr.144899.112	1	1
34850108	10.1093/nar/gkab1100	1	1
32133495	10.1093/nar/gkaa147	1	1
37516102	10.1016/j.celrep.2023.112840	1	1
31164647	10.1038/s41467-019-10439-y	1	1
35082832	10.3389/fgene.2021.785934	1	1
31464371	10.15252/msb.20198875	1	1
34390653	10.1016/j.ajhg.2021.07.009	1	1
29728462	10.1073/pnas.1722055115	7	1
31503409	10.1002/ajmg.b.32761	1	1
30537984	10.1186/s13059-018-1589-8	1	1
27831498	10.1101/gr.212092.116	1	1
37879864	10.1261/rna.079752.123	1	1
38442181	0.1073/pnas.2309469121	1	1
33179598	10.7554/eLife.62669	1	1
36947129	10.7554/eLife.83593	1	1
37906604	10.1371/journal.pgen.1011014	1	1
27259154	10.1016/j.cell.2016.04.048	1	1
34663436	10.1186/s13059-021-02509-6	1	1
38997252	10.1038/s41467-024-50174-7	1	1
40393459	10.1016/j.xgen.2025.100882	1	1
27078102	10.1073/pnas.1602886113	2	1
31344026	10.1371/journal.pgen.1008287	1	1
31227602	10.1101/gr.245159.118	1	1
25340400	10.1371/journal.pgen.1004592	1	1
27783940	10.1016/j.celrep.2016.09.066	2	1
38177677	10.1038/s41594-023-01171-9	1	1
32043966	10.7554/eLife.41279	1	1
33046894	10.1038/s41592-020-0965-y	1	2
34475398	10.1038/s41467-021-25514-6	1	1

35315433	10.1038/s41467-022-28659-0	1	1
36834916	10.3390/ijms24043509	1	1
23328393	10.1126/science.1232542	1	1
23892608	10.1038/ng.2713	1	1
25872643	10.1038/ncomms7905	1	1
26486725	10.1101/gr.191593.115	1	1
26576614	10.1101/gr.193789.115	1	1
27311442	10.1101/gr.204834.116	1	2
27524623	10.1016/j.celrep.2016.07.050	1	1
27565349	10.1016/j.cell.2016.07.049	1	1
27667684	10.1016/j.cell.2016.08.071	1	1
27701403	10.1038/nbt.3678	1	1
28137873	10.1073/pnas.1621150114	1	1
28973438	10.1093/nar/gkx577	1	1
29061142	10.1186/s13059-017-1322-z	1	1
29151363	10.1186/s13059-017-1345-5	1	1
29256496	10.1038/nmeth.4534	2	1
29789573	10.1038/s41467-018-04451-x	1	1
30045748	10.1186/s13059-018-1473-6	1	1
30568279	10.1038/s41467-018-07746-1	1	1
31267113	10.1038/s41587-019-0164-5	1	1
31395865	10.1038/s41467-019-11526-w	1	1
32248749	10.1161/ CIRCRESAHA.119.316006	1	1
32483191	10.1038/s41467-020-16590-1	1	1
32616518	10.1101/gr.260463.119	1	1
32747698	10.1038/s41398-020-00953-9	1	1
39848247	10.1016/j.cell.2024.12.022	1	1
39443793	10.1038/s41586-024-08070-z	1	1
38378865	10.1038/s41588-024-01669-y	1	1
39317738	10.1038/s41588-024-01896-3	1	1
39644900	10.1016/j.cels.2024.11.003	3	1
38724816	10.1007/s10517-024-06074-3	1	
38947339	10.3389/fimmu.2024.1387253	1	1
39631147	10.1016/j.ebiom.2024.105480	1	1
38773080	10.1038/s41467-024-48436-5	1	1
39609378	10.1038/s41467-024-54502-9	3	1
39738051	10.1038/s41467-024-55274-y	1	1
40205616	10.1186/s13073-025-01459-z	1	1
40846081	10.1016/j.jaci.2025.07.032	1	1
38413607	10.1038/s41531-024-00659-5	1	1
40680142	10.1126/sciadv.ads9164	1	1
38334359	10.7554/eLife.85235	1	1
39532105	10.1016/j.devcel.2024.10.017	1	1
40670354	10.1038/s41467-025-61734-w	1	1
40494627	10.1101/gr.280320.124	1	1
39995040	10.1093/nar/gkaf097	1	1
40414878	10.1186/s13059-025-03610-w	2	1
40715118	10.1038/s41467-025-62000-9	1	1
40738258	10.1016/j.jgg.2025.07.008	1	1

40838804	10.1093/g3journal/jkaf192	1	1
39737967	10.1038/s41467-024-54723-y	1	1
40586305	10.1093/nar/gkaf568	2	1
40399339	10.1038/s41467-025-60023-w	1	1
40318978	10.1016/j.ard.2025.04.001	1	1
39964837	10.7554/eLife.97682	1	1
40485594	10.1093/plcell/koaf084	1	1
40210244	10.1093/nar/gkaf224	1	1
40659498	10.1101/gr.279957.124	1	1
40274775	10.1038/s41467-025-58970-5	1	1
38997781	10.1186/s12920-024-01954-z	1	1
