# Supplementary material for: Massively parallel reporter assay reveals promoter-, position-, and strand-specific effects in transcription start sites
Source: bioRxiv. 2025 Oct 14:2025.10.13.659964. Preprint. [Version 1] doi: 10.1101/2025.10.13.659964 (PMC12632999; doi:10.1101/2025.10.13.659964)
Supplement: Supplement 2 [file media-2.pdf]

Supplementary Data 3: Coefficients from elastic net model predicting promoter bias from overlap with transcription-factor binding site motifs. A more positive coefficient indicates that fragments with the motif have higher plasmid activity with the SCP promoter, while a more negative coefficient indicates that fragments with the motif have higher plasmid activity with the *INS* promoter.

**Model coefficient**	**Transcription Factor Binding Site Motif**
0.319964616859408	BHLHE22_1
0.293524388458332	TEF_1
0.234710461985691	E2F_known18
0.204263463417694	ZBTB6_1
0.19603984934424	GMEB2_2
0.186138890545583	VENTX_2
0.182271895151008	`NFE2L1::MAFG_1`
0.180608817004959	HOXD13_3
0.176309707596735	NR1H4_3
0.170745518047966	ETS_known7
0.157091898449797	FOXJ2_2
0.149301928977088	PBX1_1
0.137504780183651	E2F_known1
0.129331877976445	DMRT1_1
0.122881725560097	GMEB2_3
0.110419370453192	`NKX2-5_3`
0.0908973985377944	EGR1_known9
0.0890163322533468	ETS_known13
0.0882374313481226	YY1_known1
0.0795614493185918	TCF7L2_known1
0.0762473460268454	`DDIT3::CEBPA_1`
0.0744338700514282	HINFP_1
0.0653258211672438	J4153
0.0652873796625112	FEV_1
0.0630307763011365	ZEB1_known4
0.0542448861045126	ETV7_1
0.0537007683406604	SOX17_2
0.0502524385823001	NRF1_known2
0.0428308477687616	YY2_1
0.0427824552663358	CPHX_1
0.038493459562817	HIC1_5
0.0365303915255531	CUX1_6
0.0330455322662289	YY1_known4
0.0326563585595943	IRF_known16
0.0325499881255523	TBX5_5
0.0314920344104799	ZNF219_1
0.026489420955191	FLI1_4
0.026085080337118	ITGB2_1

0.024932372411848	ZNF143_known2
0.0234500185697827	TBX21_6
0.0233745362309738	PAX2_1
0.0232530391387783	KLF13_1
0.0207734236000009	HNF4_known26
0.0194351370098085	PPARA_1
0.0138849535038736	E2F_known2
0.0137622162484968	ZNF8_1
0.012743882832353	RXRG_3
0.011653148498354	AHR_2
0.0102925479948754	SOX1_1
0.00687872900643944	SMAD4_1
0.00664429233093375	ETS_known1
0.00530858741688046	E2F_known23
0.000210671864537499	E2F_known15
-0.00426912707676313	MYBL2_1
-0.00652962562299895	HSFY2_2
-0.0118848056021666	RREB1_2
-0.0242123397554972	GTF2I_1
-0.0259329693880413	SREBP_known4
-0.0278121794986198	MEF2_known1
-0.0516010887401002	SRY_6
-0.05519622820781	MZF1_2
-0.0566318611088047	SREBP_known2
-0.0703110172293828	INSM1_1
-0.0732129653287389	NR5A2_1
-0.0773233738465818	TFCP2_5
-0.0821503790409992	IRF_known4
-0.0887572281668028	HNF1_3
-0.100996617243515	RFX5_known3
-0.191864837828888	POU6F1_3
-0.243220201327591	MSX1_1
-0.35691978758685	HMBOX1_2
-0.412821128735226	RUNX1_4
-0.668489908611629	FOXO3_4
