# Supplementary material for: Massively parallel reporter assay reveals promoter-, position-, and strand-specific effects in transcription start sites
Source: bioRxiv. 2025 Oct 14:2025.10.13.659964. Preprint. [Version 1] doi: 10.1101/2025.10.13.659964 (PMC12632999; doi:10.1101/2025.10.13.659964)
Supplement: Supplement 3 [file media-3.pdf]

Supplementary Data 2: Coefficients from elastic net model predicting position bias from overlap with transcription-factor binding site (TFBS) motifs. A more positive coefficient indicates that fragments with the motif have higher plasmid activity in the upstream configuration, while a more negative coefficient indicates that fragments with the motif have higher plasmid activity in the downstream configuration.

**Model coefficient**	**Transcription Factor Binding Site Motif**
3.52562996176848	BHLHE22_1
3.01497004058349	FOXJ2_2
1.46373927636467	YY2_1
1.39376099466038	NR2E1_1
1.37499562178328	ETS_known13
1.33562589031284	E2F_known1
1.18321296532986	ETV7_1
1.111182767387	ETS_known7
1.0603185394601	GMEB2_3
0.834796942353679	SPDEF_1
0.79638668598655	ZNF143_known2
0.749971766187846	`NKX2-5_3`
0.72463559360545	VENTX_2
0.680933478499298	NRF1_known2
0.658746625595083	ZNF8_1
0.618219196152637	E2F_known18
0.614670634596061	YY1_known4
0.610346064006302	FEV_1
0.605373454050388	T_2
0.560754949733233	HINFP_1
0.536288449402198	RORA_2
0.522227354525412	E2F_known2
0.496979582480918	FOXB1_1
0.469412507538722	YY1_known1
0.383538162664215	E2F_known23
0.337682023833318	NR3C1_known13
0.318763350664279	GMEB2_2
0.310562017318377	RARG_8
0.274213277970278	GLI2_2
0.227435738335378	IRF_known2
0.217991874333012	ETV6_1
0.211273996625631	ARNTL_1
0.208619890638352	HIF1A_1
0.187387875012351	OSR2_1
0.151413925419386	SMAD_1
0.151064668557514	IRF_known10
0.146908595490574	EGR1_known9

0.130150171843634	CUX1_6
0.113668922939779	ATF3_known5
0.0978633071316993	NR1H4_3
0.0753074223720239	SP4_2
0.0670299999647747	GFI1_1
0.0649585846780402	SPI1_known1
0.0417198905083158	RORA_7
0.0406914524636692	IRF_known16
0.0328258673258894	KLF13_1
0.0140713940816713	HNF4_known26
0.000191995292464116	EN1_1
-0.00345412472045617	IRF_known6
-0.00530653286737288	`AHR::ARNT_1`
-0.0763846986188357	GRHL1_1
-0.218241139180648	NR1H4_1
-0.220717965405663	MYB_2
-0.244008651051658	RREB1_1
-0.330204290935185	E2F7_1
-0.337958969051004	ZEB1_known1
-0.451622299464286	NFIC_1
